# Role of Vascular Smooth Muscle Cell Phenotype Switching in Arteriogenesis

**DOI:** 10.3390/ijms221910585

**Published:** 2021-09-30

**Authors:** Jasni Viralippurath Ashraf, Ayman Al Haj Zen

**Affiliations:** College of Health & Life Sciences, Hamad Bin Khalifa University, Qatar Foundation, Doha 34110, Qatar; JAshraf@hbku.edu.qa

**Keywords:** vascular smooth muscle cell, phenotypic switch, arteriogenesis, collateral arteries, peripheral arterial disease

## Abstract

Arteriogenesis is one of the primary physiological means by which the circulatory collateral system restores blood flow after significant arterial occlusion in peripheral arterial disease patients. Vascular smooth muscle cells (VSMCs) are the predominant cell type in collateral arteries and respond to altered blood flow and inflammatory conditions after an arterial occlusion by switching their phenotype between quiescent contractile and proliferative synthetic states. Maintaining the contractile state of VSMC is required for collateral vascular function to regulate blood vessel tone and blood flow during arteriogenesis, whereas synthetic SMCs are crucial in the growth and remodeling of the collateral media layer to establish more stable conduit arteries. Timely VSMC phenotype switching requires a set of coordinated actions of molecular and cellular mediators to result in an expansive remodeling of collaterals that restores the blood flow effectively into downstream ischemic tissues. This review overviews the role of VSMC phenotypic switching in the physiological arteriogenesis process and how the VSMC phenotype is affected by the primary triggers of arteriogenesis such as blood flow hemodynamic forces and inflammation. Better understanding the role of VSMC phenotype switching during arteriogenesis can identify novel therapeutic strategies to enhance revascularization in peripheral arterial disease.

In peripheral arterial disease (PAD), atherosclerosis limits blood flow to the lower extremities and represents approximately 25% of the global burden of cardiovascular disease and 1.7% of the overall global burden of disease [1]. In people over 50 years of age, 40–50% will manifest atypical symptoms in lower extremities, 10–35% with classic intermittent claudication, and 1–2% with threatened limb amputation [2,3]. The current PAD prevalence figure is expected to increase with age. The current clinical guidelines suggest to manage PAD patients’ control of cardiovascular risk factors through lifestyle modifications and vasodilators such as cilostazol to improve symptoms [4,5]. Patients who have symptoms not adequately controlled medically receive interventional endovascular treatment options or open surgery to revascularize and restore blood flow [6]. However, many patients are poor candidates for these interventions due to their comorbidities such as diabetes despite the availability of treatment options [7]. Moreover, 20% of treated patients develop recurrent symptoms that cannot further undergo vascular intervention [8,9]. Thus, the induction of endogenous revascularization has been attempted to establish an alternative strategy of revascularization that ensures efficient re-establishment of blood flow to ischemic tissues and improve PAD patients’ clinical outcomes.

The endogenous revascularization after ischemic insult involves multiple biological processes including vasculogenesis, angiogenesis, and arteriogenesis. While vasculogenesis occurs mainly during embryonic life, experimental evidence has shown that vasculogenesis contributes to adult neovascularization at least to repair the damaged capillary networks. In vasculogenesis, blood vessels form de novo via the differentiation of progenitor vascular cells into discrete vascular cells such as endothelial cells [10,11], smooth muscle cells (SMCs) [10], and pericytes [12]. Angiogenesis is a more effective blood vessel formation process in adults and is stimulated by the hypoxic inflammatory environment following arterial occlusion [13,14]. It involves sprouting new blood vessels from pre-existing capillaries to repair the vascular damage and restore tissue perfusion in distal ischemic tissues [15,16]. However, both vasculogenesis and angiogenesis are natural adaptive processes that do not bypass the blocked arteries instantly but require a long time to build a functional vascular network infiltrating ischemic tissues [17]. At the same time, innate collateral circulation mediates a quick adaptive response. Collaterals are the cross-connecting anastomoses between two feed arteries or crowns of adjacent arterial trees [18]. They are functionally and phenotypically different from arteries and veins as the blood flow along their length comes from opposite directions in the healthy tissue at baseline [19]. While significant variability exists between individuals and species in the extent of the pre-existing collateral trees [20,21,22,23], overwhelming findings demonstrate that these vascular tree arrangements can function as a natural bypass following significant arterial occlusions regardless of the extent of the pre-existing collaterals. That compensation is provided by dilating and enlarging these pre-existing vessels: this process is described as “arteriogenesis” [24,25]. Humans have progressive enlargement of pre-existing genicular arteries as collaterals from 2 to 8 weeks after femoral artery occlusion [26].

Nevertheless, most of our knowledge about the pathophysiology of arteriogenesis is based on experimental animal studies of arterial occlusion. The terms collateralization and arterialization are often confused with arteriogenesis and are poorly defined [19]. This review focuses on the arteriogenesis of collaterals as an arterial wall remodeling process where the pre-existing collaterals grow with an increase in diameter and wall thickness in response to an initial hemodynamic stimulus [27].

## 1. The Pathophysiology of Arteriogenesis after Ischemia

When a primary arterial trunk is occluded, it leads to a pressure drop downstream of the arterial network subsequently creating a pressure gradient across pre-existing collateral circulation and forcing the diversion of blood flow through the collaterals. The altered blood flow generates hemodynamic forces in collateral arterioles and arteries triggering two vascular responses: short-term vasodilation and long-term expansive vascular remodeling [28]. The short-term phase mediated by vasoactive molecules such as nitric oxide relax the smooth muscle cells leading to vasodilation [29]. The acute alteration of fluid shear stress induces the expression of chemokines and adhesion molecules by the endothelium [30]. Chemokines trigger the recruitment and attachment of circulating monocytes to activated endothelium that express adhesion molecules [31]. Monocytes transmigrate through the endothelium into the sub-intimal space where they transform into macrophages and produce inflammatory cytokines and growth factors such as transforming growth factor-β (TGFβ) [32], tumor necrosis factor-α (TNFα) [33], epidermal growth factor (EGF) [34], and fibroblast growth factor (FGF) [35]. The accumulation of macrophages has been reported in the perivascular area as well [36]. Eventually, these cytokines and growth factors diffuse into the medial layer of collaterals modulating the signaling pathways of SMC [37]. This results in SMC phenotypic switching that involves the SMC dedifferentiation from the quiescent contractile phenotype to a proliferative, migratory, and synthetic phenotype [38]. Growth factors and cytokines secreted from macrophages activate the proteolysis system for extracellular matrix (ECM) remodeling to further enhance SMC phenotypic switching [39]. 

Synthetic smooth muscle cells migrate from the media to the subendothelial space (intima) where they proliferate abundantly and produce ECM components including collagen, elastin, and proteoglycans to the subintimal space resulting in the formation of a new layer of SMC [38]. At this point, the collateral vessel has an approximately 25-fold larger diameter with a newly formed tunica intima, reconstituted tunica media, and thickened tunica adventitia that can restore blood flow up to 50% [40]. The increased vascular diameter is associated with the normalization of shear stress and mechanical strain on the vascular wall [41]. This reduced intravascular pressure in the collaterals impairs endothelial activation, attenuates inflammation, and causes synthetic SMCs to re-differentiate back into their contractile state, thus terminating the collateral growth process [42]. Concurrently, blood flow is reduced and gradually regresses in collaterals that fail to have mature arteriogenesis [43]. 

**Figure 1:**
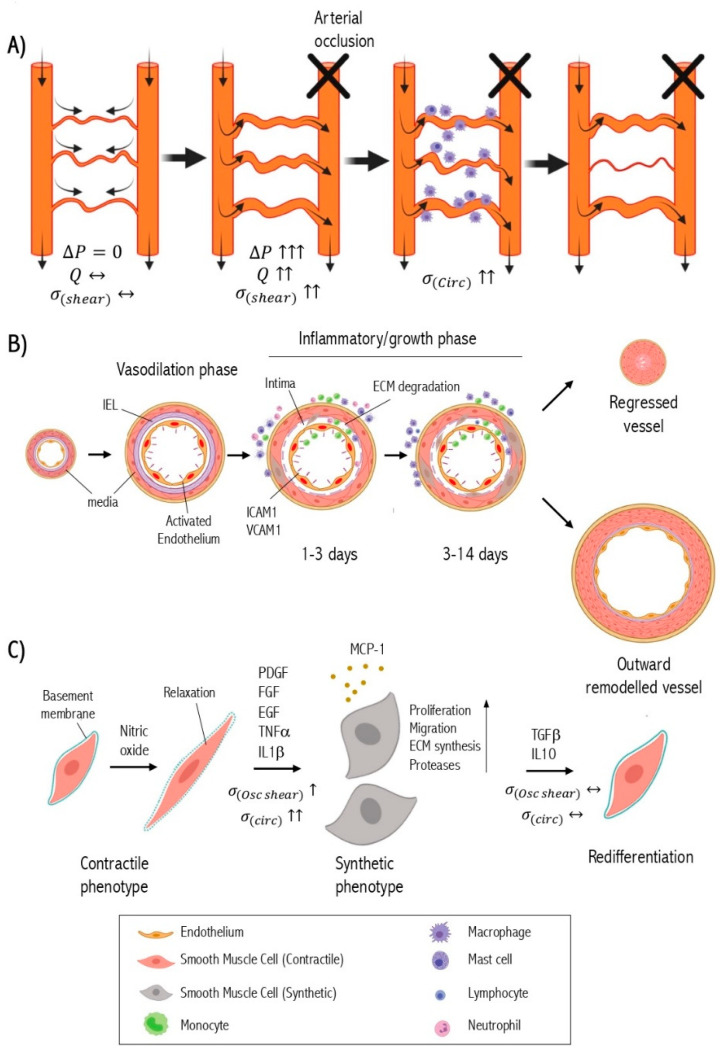
Pathophysiology of arteriogenesis. (**A**) Diagram illustrates the hemodynamic changes of collateral circulation after arterial occlusion. Pressure-gradient (ΔP); blood flow (Q); direction of blood flow (arrows); fluid shear stress (σ_(shear)_); circumferential wall stress or tension (σ_(circ)_). (**B**) Cross-section of collateral arteries showing the different phases of the physiological arteriogenesis (IEL: internal elastic lamina). The presented time course of arteriogenesis phases is observed in the mouse model of limb ischemia. (**C**) The dynamics of the vascular smooth muscle cell (VSMC) phenotype switch during arteriogenesis. Oscillatory shear stress (σ_(shear)_). The figure has been created with BioRender.com.

Upon successful arteriogenesis, the collaterals exhibit an extensive outward and hypertrophic remodeling, which is associated with the transformation of a small microvascular resistance vessel into a large conductance artery [44]. The smooth muscle cell is the primary cell type in this collateral remodeling [45] (Figure 1).

## 2. Molecular Regulation of SMC Phenotype Switching

Vascular smooth muscle cells in the adult vasculature are not terminally differentiated cells. They possess extensive plasticity such that it can be stimulated to undergo a structural and functional transition into proliferative/migratory/synthetic phenotype or undergo an extreme phenotypic change into osteochondrocyte-like cells [46], foam-like cells [47], and myofibroblasts [48] as detected in atherosclerotic lesions. Nevertheless, SMC plasticity enables de-differentiated SMCs to re-differentiate back to a quiescent and contractile state according to their microenvironment [49]. Many environmental factors of SMC phenotype are identified such as growth factors, cytokines, hormones, blood flow shear stress, cell-to-cell interactions, and cell-to-matrix interactions. SMCs phenotypic switching is a critical event in the pathogenesis of arterial wall diseases such as atherosclerosis [50], aneurysm [51], hypertension [52], and post-angioplasty restenosis [53].

The mature contractile phenotype of SMCs is morphologically characterized by low numbers of protein synthesis organelles, e.g., rough endoplasmic reticulum, Golgi apparatus, or free ribosomes [54]. They demonstrate high expression of proteins that are involved in muscle contraction and anchorage including α-smooth muscle actin (αSM actin) [55], smooth muscle-myosin heavy chain (SM-MHC) [56], h1-calponin [57], smooth muscle 22α (SM22α) [57], and smoothelin [58]. In contrast, synthetic SMCs have little or no contractile protein content and high active protein synthesis apparatus [59]. Synthetic SMCs produce pro-inflammatory factors such as TNF-α [60], C-C Motif Chemokine Ligand 2 (CCL2) or monocyte chemoattractant protein-1 (MCP-1) [61] and ECM remodeling proteins such as collagen I [62] and matrix-metalloproteinases [63]. Most of these proteins are involved in tissue repair and remodeling, which reflect the functional role of synthetic SMCs. Synthetic phenotypes are associated with abnormal mechanical forces.

The molecular basis of sustaining the SMC contractile state has been explained by maintaining CArG–SRF–Myocardin complex [64]. Any headway to disrupt this complex leads to down-regulation of genes encoding for contractile proteins, thus inducing a phenotypic switch to a synthetic pathways [65]. Indeed, the expression of the contractile genes is controlled by multiple CArG elements located within their promoter-enhancer regions [66]. The transcription factor serum response factor (SRF) binds to a general sequence motif in the CArG element (CC(A/T-rich)6GG) to regulate the expression of marker genes [67]. Myocardin (MYOCD) is a potent coactivator of SRF and acts as a mediator of environmental cues on the expression of SMC contractile genes [68]. Myocardin-related transcription factors (MRTFs) [69] and ternary complex factors (TCF) [70] have also been identified to be cofactors of SRF. Prior work has shown that MYOCD and MRTFs respond to pro-differentiation stimuli through Rho GTPases-actin signaling [71,72], whereas TCFs respond to dedifferentiation stimuli through mitogen-activated protein kinase (MAPK) signaling [73]. 

Several pathways transduce signals from cell surface receptors or integrins in response to the surrounding environmental factors to maintain the contractile phenotype. The RhoA/ROCK signaling triggers actin polymerization increases post-translational modification of MRTFs and releases it to the nucleus to induce contractile gene expression [69]. TGFβ enhances nuclear translocation of SMAD proteins. Interaction with SMAD-binding elements (SBEs) in turn upregulates the expression of differentiation marker genes [74]. Insulin growth factor (IGF) acts through PI3 K/AKT signaling which relieves FOXO4-repressive effect on the CArG–SRF–myocardin complex leading to stable expression of contractile genes [75,76].

**Figure 2:**
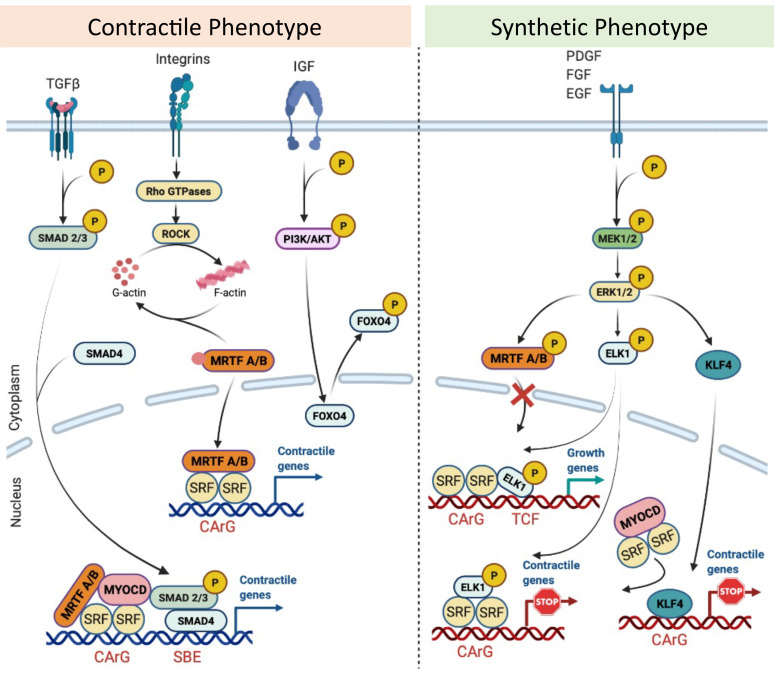
Molecular regulation of vascular smooth muscle phenotype. TGFβ induces the formation of pSMAD2/3-SMAD4 complex, which is translocated into the nucleus, where it binds to SMAD-binding elements (SBE), leading to the expression of early contractile SMC genes. The SMAD interaction with myocardin (MYOCD) or MRTFs enforces SMC differentiation and maturation. Rho/ROCK activates the actin polymerization, which release G-actin monomers from MRTFs, enabling MRTFs to translocate into the nucleus. There, they bind to the SRF/CArG, inducing the expression of SMC contractile genes. IGF activates the PI3 K/AKT signaling pathway that phosphorylates the nuclear FOXO4 facilitating the nuclear export of FOXO4 to release the repression on the CArG/SRF/MYOCD complex. Growth factors (e.g., FGF, PDGF, EGF) through the MEK/ERK pathway represses SMC contractile genes by phosphorylation of the ternary complex factor (TCF) Elk-1 and MRTFs, and by increasing KLF4 level. Phospho-Elk-1 abolishes the SRF interaction with MYOCD or induces SRF-dependent transcription of early growth genes. The phosphorylation of MRTFs prevents their translocation into the nucleus. The figure has been created with BioRender.com.

The loss of the contractile phenotype occurs via growth factors such as Platelet-derived growth factor-BB (PDGF-BB), FGF, and EGF that activate MAPK cascade via the Ras/Raf/MEK/ERK pathway. The MAPK activation can phosphorylate and activate TCF proteins such as Elk-1 to displace MYOCD or induce SRF-dependent transcription of early response growth, dedifferentiation genes, and repression of smooth muscle contractile genes [77]. ERK can phosphorylate MRTFs in the cytoplasm and prevent nuclear translocation [78]. PDGF-BB induces Kruppel Like Factor 4 (KLF-4) by binding to G/C repressor elements or by competing with SRF for CArG elements to disrupt CArG–SRF–myocardin [79,80] (Figure 2). Epigenetic regulation has been reported to control SMC phenotype switching [81]. For instance, the overexpression of histone acetyltransferase (HAT) enhances TGF β1-regulated SMC marker gene expression and its inhibitors such as Twist1 and E1A. Histone deacetylase (HDAC) expression converses this effect of TGF β1 in SMC [82]. Decreased DNA methyltransferase activity and DNA hypomethylation was observed in the proliferating intimal SMC present in the atherosclerotic lesions both in vivo and in vitro [83,84]. Although some recent advancement sheds light on to the influence of environmental cues in modifying epigenome, deeper research involving genome-wide profiling with epigenetic markers are warranted to completely understand its role in SMC phenotype switching in arteriogenesis. 

## 3. The Effect of Hemodynamics on Smooth Muscle Cell Phenotype during Arteriogenesis

Upon arterial occlusion, the increase of blood flow and intravascular pressure in the bypass collaterals can generate two primary forces: fluid shear stress and circumferential wall tension [43]. The fluid shear stress force directly affects the endothelium [85]. Circumferential wall tension affects both endothelium and medial smooth muscle cells [86]. However, the fluid shear stress can indirectly affect the smooth muscle cells through diffusible vasoactive molecules secreted from activated endothelium, inflammation-induced shear stress factors, and shear stress generated by secondary interstitial flow [87]. Multiple in vitro studies have shown that both generated physical hemodynamic forces can influence the SMC phenotype [85]. Under physiological conditions, collaterals typically have little or no blood flow, and the fluid shear stress is minimal with little influence on the SMC phenotype [19]. Here, the phenotype of contractile SMCs in the collaterals is maintained mainly by the kinetic energy of flow, which is converted to potential energy that maintains the high circumferential wall stress of collaterals [88]. This also leads to an intense SMC investment of the collaterals unlike distal arterioles.

In contrast, after arterial occlusion, early vasodilation of collaterals leads to a rapid decrease in the intra-luminal pressure. Thus, according to Laplace’s formula, we expect that the circumferential wall stress would not change significantly in the early stage. In addition, the diminished blood pressure in downstream vessels is much lower than the proximal arterial pressure; thus, this pressure is unlikely to cause a significant stretch force on medial SMC at this phase. Indeed, the SMC in small arteries and arterioles generally react to the acute rise of intraluminal pressure by contraction (myogenic response) to regulate the tissue blood flow [89,90]. However, the collaterals are dilated after the arterial occlusion indicating that shear-mediated responses predominate the intravascular pressure-mediated responses or this could be partially explained by the fact that collaterals lack myogenic responsiveness and have less SMC tone at baseline than the arteries/arterioles they interconnect [88]. Endothelial-smooth muscle cell co-culture studies have shown sustained exposure of endothelial cells to laminar shear stress inhibits SMC proliferation and induces a transition from a synthetic to a contractile phenotype [91]. These observations agree with in vivo animal studies showing an absence of SMC proliferation of the SMC phenotype switching at early stage (up to 48 hours after arterial occlusion) [92]. In summary, the increase in fluid shear stress is the dominant factor at the very beginnings where the SMCs are relaxed while maintaining their contractile phenotype.

The increase in fluid shear stress force is transmitted to the SMC by diffusible molecules such as nitric oxide (NO) [93]. NO is synthesized by endothelial nitric oxide synthase (eNOS) or inducible nitric oxide synthase (iNOS) [94]. NO can diffuse into the smooth muscle cells where it binds with soluble guanylate cyclase (cGC) to produce cyclic guanosine monophosphate (cGMP), which is a crucial factor to activate protein kinase G (PKG) that relaxes the smooth muscle cells leading to vasodilatation [95]. NO inhibits SMC proliferation through the extracellular signal-regulated kinase (ERK) pathway leading to increased protein levels of the cyclin-dependent kinase inhibitor p21 Waf1/Cip1 [96]. cGMP-dependent protein kinase (PKG) overexpression in synthetic SMC results in phenotypic switching of SMC to a contractile phenotype that expresses contractile markers [97] (SM-MHC, calponin, α-SM actin) with reduced expression of synthetic phenotype markers (osteopontin, thrombospondin) [98,99,100,101]. Despite the continuous production of NO through the process of arteriogenesis, the effect of NO on SMC phenotype is not predominant in the next stages of arteriogenesis and is overridden by other factors such as cytokines, growth factors, and the increase of circumferential wall tension. All these factors tend to induce the SMC synthetic phenotype. Mees et al. used the distal femoral artery ligation model that only causes marginal ischemia in the lower limb to specifically study arteriogenesis. Their findings demonstrate that tissue blood flow recovery was impaired in eNOS-knockout mice due to the inability to sufficiently vasodilate collaterals and not because of impaired arteriogenesis [102]. These findings were supported by several studies using mathematical modeling that showed that collateral vasodilation is a critical triggering factor for significant blood flow compensation to occur following arterial occlusion [103,104]. 

Most collaterals tend to be tortuous small arteries [105]. The steady low shear stress is transformed rapidly to turbulent shear stress when blood flow increases after proximal arterial occlusion. The sudden changes in fluid shear stress naturally promote acute activation of endothelial cells and inflammatory pathways [106]. Endothelial cells sense the change of fluid shear stress via mechanosensors and covert this into paracrine chemical signals that influence the medial SMC [107]. The mechanosensation process involves multiple endothelial cell components. For instance, the activation of volume-regulated endothelial chloride channels is one of the earliest responses to endothelial cell swelling resulting from an acute increase in fluid shear stress [108]. Other early responses led to mechanically gated channels such as transient receptor potential cation channel V4 (TRPV4) and Piezo1. These are sensitive to changes in the endothelial cell membrane’s tension resulting from fluid shear stress [109].

Endothelial surface glycocalyx and its components can act as a mechanoreceptor and can transmit mechanical stimuli to the cytoskeleton, which can then activate downstream signaling pathways such as PI3K/AKT/eNOS and NFκB [110]. The platelet endothelial cell adhesion molecule-1 (PECAM-1), vascular endothelial-cadherin (VE-cadherin), and vascular endothelial growth factor receptor 2 (VEGFR2) can collectively act as mechanosensory complex in response to the fluid shear stress [111]. In vivo, the genetic deletion of the PECAM-1 component attenuates the NF-kB activation and downstream inflammatory response in collateral arteries following limb ischemia [112]. This was associated with partial recovery of blood flow and reduced collateral remodeling. 

In contrast, the alterations in the intraluminal pressure in the later stages of arteriogenesis can be extended to exert a mechanical force on the cellular components of VSMCs, which act as a mechanosensor to initiate subsequent signal transduction events [43]. Medial SMC can also be indirectly exposed to the fluid shear stress and blood flow pressure through the (transmural) interstitial flow. This flow shear stress is driven by the transmural pressure differential between the intra-arterial and tissue pressure [113] and can exhibit a direct shear stress force on SMCs that their mechanosensors can sense. Many mechanosensors were identified on VSMCs to sense the surrounding mechanical stimuli such as membrane-like receptors, ion channels and pumps, glycocalyx, primary cilium, and integrins [114,115]. These mechanosensors could transmit signals from the surroundings to affect the SMC phenotype as an adaptive response [116]. Hu et al. demonstrated that the adaptor molecules of membrane mechanosensors are inactive in the quiescent SMC. The altered mechanical stress initially induces a conformational change in the plasma membrane leading to autophosphorylation of PDGFα receptors and sequential activation of MAPK cascades [117].

Additionally, activation of integrin receptors, stretch-activated cation channels, and G proteins are also observed in SMC membranes of collateral vessels in response to stretch forces [118]. These forces play a pivotal role in SMC proliferation and differentiation [117]. SMCs are aligned circumferentially in the media layer. SMCs stretch along their central axis when the collaterals vasodilate due to the hemodynamic changes. The vasodilation might elevate circumferential wall stress via thinning of the pressure-bearing vessel wall leading to increased SMC wall mass as negative feedback of circumferential wall stress regulation [119]. This process requires VSMC proliferation, matrix degradation, and migration, which drives the ability to sense and adapt to mechanical stresses. In vitro studies have demonstrated that cyclic stretching activates ERK1/2 signaling bringing down the expression of SM-MHC, smoothelin, and calponin in VSMCs [120]. Downregulation of SMC marker genes mediates phenotypic modulation and sustained phosphorylation of ERK1/2 and contributes to the SMC medial layer’s growth during arteriogenesis [121]. 

Cyclic stretching is a critical inducer of MCP-1 expression in SMC of remodeling collaterals [122]. It governs the recruitment of circulating monocytes and stimulates SMC’s proliferation and inflammatory state through differential activation of the transcription factors NF-kB and AP-1 [123]. Ephrin B2 is another well-known inducer of arteriogenesis and is controlled by cyclic stretch. Ephrin B2 is an arterial marker that is upregulated in endothelial cells during collateral remodeling and plays a vital role in arteriogenesis by limiting SMC migration within defined borders and controlling monocyte extravasation [124]. Exposure of SMCs to cyclic stretch also increases collagen and fibronectin production, metalloproteinase activity, and TGFβ expression, thus modulating the arterial remodeling outcome [125].

The arterial wall strain is chronically elevated in systemic hypertension conditions. The small arteries and arterioles remodel inwardly through a eutrophic process of rearrangement of the same SMC around a smaller lumen [126]. Conversely, collateral arteries undergo robust anatomic outward remodeling [127]. The inflammatory profile plays a pivotal shift in the remodeling outcome towards expansive remodeling in response to the initial shear stress changes after arterial occlusion. There is a tight association between fluid shear stress and wall stretch dynamics according to a theoretical model simulating hemodynamic alterations-stimulated vascular remodeling responses [128]. Maintaining the relationship between the two forces is regarded a design principle for adequate collateral circulation [41].

## 4. The Role of Inflammation in SMC Phenotypic Change

The initial phase of collateral vasodilation is driven by fluid shear stress and occurs within the early stage of post-occlusion. However, this vessel enlargement accounts for a small proportion of final vessel expansion, and it slows following shear stress normalization [85]. The appearance of synthetic SMCs or a reduction in contractile SMCs occurs after the vasodilation phase in coincidence with increased inflammatory intensity. The inflammatory process is triggered by shear stress alterations and is an amplifying factor that drives the vessel expansion beyond this point to influence SMC phenotype [129]. Similar to the formation of atherosclerotic plaque in which inflammation sought to play an important role in SMC phenotypic modulation [130], a complex inflammation process leads to cell-to-cell crosstalk between VSMCs, endothelial cells, and immune cells for their phenotypic transition in the context of collateral remodeling [131]. The shear stress-induced inflammation offers a beneficial effect on arteriogenesis only if it remains transient. Indeed, that prolonged inflammation leads to ineffective arteriogenesis [132]. Hence, the factors affecting the time required to trigger inflammation must be well evaluated because the variability in individuals accounts for the variation in the extent of arteriogenesis between individuals and species.

Endothelial cells in pre-existing collaterals are the first effectors to initialize an inflammatory process following arterial occlusion [133]. In response to fluid shear stress alterations, endothelial cells express cell adhesion molecules, chemokines, and cytokines. Chemokines are vital mediators that recruit circulating monocytes into the subendothelial space of collaterals [134]. Additionally, significant accumulation of monocytes/macrophages in the perivascular area peaked within the first three days of arterial occlusion [31]. SMCs express many chemokine receptors on their surface, and they are responsive to accumulated chemokines in the collaterals. Therefore, chemokines can directly contribute to the modulation of SMC phenotypes during arteriogenesis. Exogenous MCP-1 is a potent chemokine in enhancing arteriogenesis and stimulates SMC proliferation [135]. In vitro forced expression of MCP-1 in SMCs leads to dedifferentiation [136]. The role of other chemokines involved in arteriogenesis and SMC phenotype changes remains unclear. 

Medial SMCs also contributes to initiating or amplifying stretch-induced inflammation in collateral remodeling [137]. They produce multiple cytokines and chemokines, e.g., interleukin IL-1, IL-6, CCL2, and C-X-C Motif Chemokine Ligand 10 (CXCL10) in response to changes in intravascular pressure [138]. However, the collateral media often remains spared from immune cell infiltration. Monocytes/macrophages can mainly regulate the SMC phenotype through paracrine effects during arteriogenesis [139]. Monocytes are divided into a subset of populations based on the expression levels of cell surface markers and chemokine receptors: pro-inflammatory (Ly6 C^high^CCR2^high^CX3 CR1^low^) and tissue repair (Ly6 C^low^CCR2^low^CX3 CR1^high^) in *mouse* [140]. The infiltrated pro-inflammatory monocytes (Ly6C^high^) are detected mainly in the collaterals during the early phase of arteriogenesis whereas the anti-inflammatory monocytes (Ly6 C^low^) predominate the collaterals during the growth and expansion phase of arteriogenesis [141]. Ly6 C^high^ monocytes are more likely to differentiate into M1 macrophages, which secrete various pro-inflammatory cytokines such as interleukin (IL)-1, IL-6, IL-12, and TNFα. They exhibit high proteolytic activity [142]. In contrast, Ly6 C^low^ monocytes may differentiate into M2 macrophages, which secrete anti-inflammatory cytokines such as IL-10 and TGFβ1 and express growth factors such as vascular endothelial growth factor (VEGF) and basic fibroblast growth factor (bFGF), thus promoting collateral remodeling and expansion [143]. Many of the factors produced by macrophages have been linked with SMC growth and loss of contractile phenotype [144,145,146]. In vitro co-culture studies have shown that macrophage-derived PDGF enhances SMC proliferation and suppresses the expression of SMC contractile markers: SM α-actin and SM-MHC. IL-6 released by macrophages promotes Matrix metalloproteinase-1 (MMP-1) production by SMC [147]. Other factors such as TGF-β1 that is secreted by the M2 macrophage induces a contractile SMC phenotype [148,149].

Monocytes and macrophages are an important source of metalloproteinases and other proteases such as cathepsins during vascular repair process [150]. During arteriogenesis, metalloproteinases contribute actively to extracellular matrix breakdown to facilitate SMC migration and rearrangement. MMP-2 and MMP-9 stimulates the interaction of VSMCs with newly formed ECM to trigger intracellular signaling via integrins to induce a phenotypic switch and persistent migration [151]. SMCs develop an intercellular signaling system de novo: connexin-37 is a highly specific marker for developing collateral vessels [152]. All of these changes contribute to the release of constraints imposed by the structural scaffold of extracellular matrix. In turn, this directs collateral remodeling towards an outward remolding. Metalloproteinases can increase the bioavailability of growth factors and cytokines by processing the bound species into an extracellular matrix, thus enhancing their capacity to regulate SMC phenotype in a spatial and temporal manner during the different phases of arteriogenesis [153]. 

Other inflammatory cell populations have been reported to infiltrate the collateral sites during arteriogenesis, e.g., neutrophils [154], mast cells [155], and lymphocytes [156]. Mast cells residing in the perivascular tissues of arteries are activated during arteriogenesis. Chillo et al. demonstrated that shear stress-induced mast cell activation is mediated by activated neutrophil and platelet-derived effectors such as reactive oxidative stress [157]. Degranulation occurs when mast cells are activated, and thus the bioactive granule content is released to the extracellular space leading to a powerful inflammatory reaction. Mast cell granules contain many bioactive constituents including vasoactive molecules, amines, cytokines, proteases, and proteoglycans that can diffuse into the media and influence the SMC function and phenotype [157]. T lymphocytes contribute to the inflammation process during arteriogenesis. Specifically, T cells positively regulate arteriogenesis as demonstrated by the impaired arteriogenesis observed in CD4+ knockout mice model for acute hindlimb ischemia [158]. Upregulation of CCR-7 and its ligands CCL19 and CCL21 were observed within 22 hours post-ischemia leading to a transient retention of CD4+ T lymphocytes in the tissue to mediate a positive role in the initial phase of arteriogenesis [132]. Additionally, the depletion of natural killer (NK) cells severely impairs arteriogenesis in C57BL/6 NK-cell-deficient transgenic mice [159]. Nevertheless, the significance of mast cells or lymphocyte role in SMC phenotype switching remains undefined in vascular pathologies including ischemia-induced arteriogenesis.

## 5. Conclusions Remarks on Targeting SMC Phenotype Switching as Therapeutic Arteriogenesis 

Arteriogenesis is a physiological remodeling response of the collateral arteries in the occlusive arterial diseases. Despite the dramatic outward remodeling of collaterals, blood flow is restored only up to 40–50% of the unblocked artery without intervention. One reason for the limited restoration of blood flow is the premature normalization of fluid shear stress (primary trigger of arteriogenesis). This natural compensatory capacity is diminished even further in subjects with co-morbid diseases such as diabetes [160]. Using an experimental shunting procedure, the induction of high continuous fluid shear stress in collateral circulation led to an increase in the maximal collateral conductance up to 80% of the maximal blood flow of the arterial tree before occlusion [161]. Indeed, this experiment is a clear proof-of-concept that a therapeutic intervention is feasible to enhance the natural arteriogenesis and overcome the anatomical and physiological limits of blood flow recovery after acute ischemia. 

Vascular smooth muscle cells play a central role during arteriogenesis due to their plasticity. Phenotypic switching of the contractile VSMC to a synthetic state is a critical cellular event that sustains the growth and outward remodeling of collaterals. In contrast, VSMC re-differentiation back to their contractile state is important to regain vascular functions and prevent inappropriate hypertrophic remodeling of collaterals that could perturb the blood flow for distal ischemic tissues. The physiological normalization of fluid shear can lead to a premature switch of synthetic VSMC to a contractile quiescent phenotype; this switch eventually attenuates the collateral wall growth and remodeling. It has been shown that growth factor therapy can stimulate angiogenesis and also is able to stimulate arteriogenesis [162,163]. Many of the used growth factors such as FGF2 and PDGF are involved in the induction of SMC phenotype switching into synthetic phenotype. However, the single growth factor therapy was never able to completely restore the conductance capacity of a larger artery [164]. Thus, administration of vasodilators combined with agents inducing synthetic SMC as therapeutic strategy could increase the maximal collateral conductance in occlusive artery disease.

Targeting vascular smooth muscle cell phenotype switching has been suggested to be a therapeutic approach for tackling other vascular diseases such as atherosclerosis and hypertension. However, since the dynamics of VSMC phenotype switching are different in arteriogenesis, the timing of intervention would be challenging to achieve effective therapy. Another challenge of targeting VSMC in arteriogenesis is that several cellular events of atherosclerotic plaque development are also involved in arteriogenesis. For instance, extracellular matrix degradation, VSMC migration, and proliferation are activated in both arteriogenesis and atherogenesis. The stimulation of arteriogenesis through agents that promote VSMC proliferation or positive arterial remodeling could have side effects on the aggravation of atherosclerotic plaques in PAD patients who typically suffer from atherosclerosis. While many experimental studies have been conducted to investigate the molecular mechanisms of VSMC phenotype regulation in the context of atherosclerosis, the molecular mechanisms of VSMC phenotype switching that specifically control arteriogenesis in ischemic vascular disease remain largely unknown. Previous studies have been carried out to trace the VSMC phenotype dynamics in vascular repair and atherosclerosis models. Further studies are required for VSMC lineage tracing studies during arteriogenesis. These can help to identify novel specific molecular targets for therapeutic arteriogenesis of peripheral arterial disease patients. 

## Data Availability

Not applicable.
